# Targeted Delivery Systems for Molecular Therapy in Skeletal Disorders

**DOI:** 10.3390/ijms17030428

**Published:** 2016-03-22

**Authors:** Lei Dang, Jin Liu, Fangfei Li, Luyao Wang, Defang Li, Baosheng Guo, Xiaojuan He, Feng Jiang, Chao Liang, Biao Liu, Shaikh Atik Badshah, Bing He, Jun Lu, Cheng Lu, Aiping Lu, Ge Zhang

**Affiliations:** Institute for Advancing Translational Medicine in Bone & Joint Diseases, School of Chinese Medicine, Hong Kong Baptist University, Hong Kong, China; danglei_hkbu@163.com (L.D.); liujin_hkbu@163.com (Ji.L.); fayebalaba@live.com (F.L.); luyaoben@126.com (L.W.); lidefang@163.com (D.L.); borisguo@hkbu.edu.hk (B.G.); hxj19@126.com (X.H.); jiangfeng@nbu.edu.cn (F.J.); liangchao512@163.com (Cha.L.); liubiao_hkbu@163.com (B.L.); aatikshaikh@gmail.com (S.A.B.); hebinghb@gmail.com (B.H.); ljaaa111@163.com (Ju.L.); lv_cheng0816@163.com (Che.L.)

**Keywords:** targeted delivery systems, skeletal disorders, targeting moieties, nanoparticles

## Abstract

Abnormalities in the integral components of bone, including bone matrix, bone mineral and bone cells, give rise to complex disturbances of skeletal development, growth and homeostasis. Non-specific drug delivery using high-dose systemic administration may decrease therapeutic efficacy of drugs and increase the risk of toxic effects in non-skeletal tissues, which remain clinical challenges in the treatment of skeletal disorders. Thus, targeted delivery systems are urgently needed to achieve higher drug delivery efficiency, improve therapeutic efficacy in the targeted cells/tissues, and minimize toxicities in non-targeted cells/tissues. In this review, we summarize recent progress in the application of different targeting moieties and nanoparticles for targeted drug delivery in skeletal disorders, and also discuss the advantages, challenges and perspectives in their clinical translation.

## 1. Introduction

The skeletal system provides a strong support and protection for the soft tissues and organs in the human body. The skeletal system includes bone matrix, bone mineral and bone cells. Any defects in these components may hinder skeletal development, growth and homeostasis, which results in skeletal disorders. The most common skeletal disorders are osteoarthritis, bone cancer, and osteoporosis. These disorders result from a wide variety of causes, some of which, such as vitamin D deficiency, are easily treatable, while the pathogenesis of some others are more complicated [1]. The drug therapy of skeletal diseases by systemic administration has many disadvantages, for example the high doses of drugs may lead to adverse effects in non-skeletal tissues and narrow down the therapeutic window [2]. Thus, there is an urgent need for therapeutic approaches to achieve higher drug delivery efficiency, improved therapeutic efficacy and minimum toxicities.

**Figure 1:**
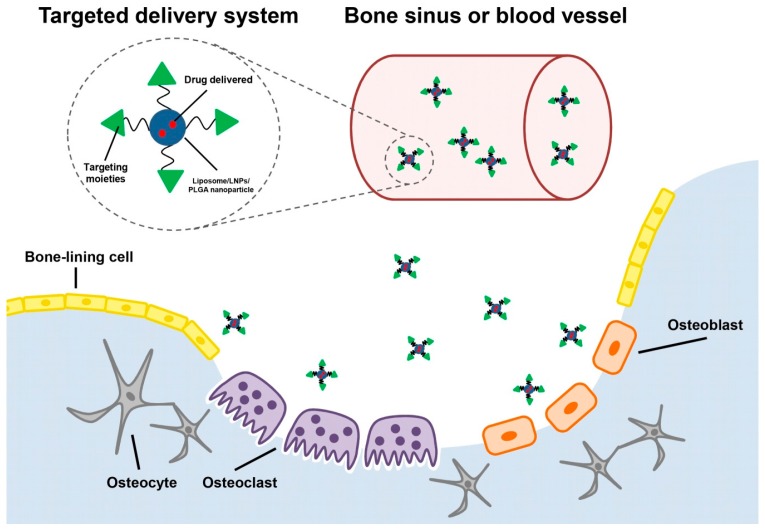
The schematic graph of targeted delivery systems carrying drugs extravasated from bone sinusoid or blood vessels to different target cells in bone. The targets for drug delivery depend on the targeting moieties in the targeted delivery systems.

One of the promising strategies is carrying drugs to specific skeletal pathological locations through targeted delivery systems. The application of targeted drug delivery offers great potential in minimizing the toxicity to non-skeletal tissues. The targeted delivery systems not only specifically deliver drugs to its desired destination, but also protect them from elimination and degradation in the blood circulation and improve solubility of the low water-soluble drugs [3,4]. Therefore, a number of targeted delivery systems used in skeletal disorders are developed or under investigation (Figure 1). To achieve potent and effective drug delivery, a suitable targeting moiety, responsible for targeting specific bone tissues or specific cell types, should be selected. To our knowledge, different types of compounds have so far been used as targeting moieties in targeted delivery systems. In addition, the most developed drug carriers used in the treatment of skeletal disorders are nanoparticles, such as liposomes, lipid nanoparticles (LNPs), and Poly(lactic-*co*-glycolic acid) (PLGA) nanoparticles. Some of nanoparticles have been also approved by the FDA (Food and Drug Administration) [5].

In this review, we mainly discuss the application of different types of targeting moieties and nanoparticles to develop targeted delivery systems and their immense clinical potential for molecular therapy in skeletal disorders.

## 2. Targeting Moieties

The lack of the specific drug delivery will increase the risk of toxicity to non-targeted tissues or cells in bone and decrease the therapeutic efficiency. Therefore, the targeting moieties could facilitate its cargo, such as drugs or drug carriers, to the targeted tissues or cells. Thus, the targeting moiety is one of the essential components in the targeted delivery systems. According to their targeting specificity, the targeting moieties can be classified into two types: bone tissue-targeting moieties, and cell-specific targeting moieties including osteoblast-targeting, osteoclast-targeting, and bone marrow mesenchymal stem cell-targeting moieties (Table 1). In the early development of targeted delivery systems, the bone tissue-targeting moieties, targeting the whole skeletal systems, are widely used. However, the biodistribution of the agents in the whole skeletal tissues results in unwanted side effects associated with its distribution to non-targeted cells. Thus, cell-specific delivery systems, attacking specific cells in bone, while doing little or no damage to normal cells, are highly desirable. This section will discuss the types of targeting moieties that are used in delivery systems for molecular therapy of skeletal disorders, as well as their potential benefits and drawbacks.

### 2.1. Bone Tissue-Targeting Moiety

The clinical hurdle to treat skeletal diseases is caused by the unfavorable adverse effects after systematic administration with high therapeutic dose of drugs. Thus, targeted delivery of drugs to bone pathological locations is an alternative strategy to overcome these problems. After decades of development, more and more different bone-seeking chemicals have been identified and used in targeted delivery systems as bone tissue-targeting moieties and can be classified into two types: synthetic compounds and biological molecules.

#### 2.1.1. Synthetic Compounds

It has been long known that the bisphosphonates and tetracycline are bone-seeking small compounds. These compounds have also been used in clinics for different therapeutic purposes, such as treating osteoporosis and inhibiting bacterial infections [17,18]. Because they have high affinity to calcium ions in bone, these compounds as targeting ligands in delivery systems could deliver their cargos or drugs to whole skeletal systems [19,20]. These targeted delivery systems could provide therapeutic strategies for pathological locations in the entire skeletal system.

##### Bisphosphonates

The bisphosphonates are a family of drugs widely used in clinics. Their mechanisms of bone tissue-targeting are well-known. Bisphosphonates are structurally similar to pyrophosphate, an endogenous regulator of calcium homeostasis. Bisphosphonates feature two germinal phosphates (P–C–P bond) in their structures instead of the P–O–P bond of pyrophosphate. This structure makes bisphosphonates not easily eliminated *in vivo*. Because the chemical structure of the P–C–P bond has high affinity to calcium crystals, the bisphosphonates are specifically targeting bone after administration intravenously or orally [21].

In addition, the bisphosphonates can affect osteoclastic functions, including osteoclast recruitment, differentiation, and resorption [22]. Due to their modulation on the calcium metabolism, bisphosphonates including pamidronate, alendronate and tiludronate have been used in clinical therapies of skeletal disorders, such as Paget’s disease, hypercalcaemia of malignancy and osteoporosis [23,24,25,26]. Thus, at the early stage of development of bone-targeted delivery systems, the bisphosphonates are ideal bone-targeting moieties with therapeutic effects on inhibition of osteoclastic bone resorption.

Alendronate, as one of the bisphosphonates family members, can coat PLGA nanoparticles encapsulating curcumin (a non-toxic multi-target chemopreventive/chemotherapeutic agent) and bortezomib (a clinically tested proteasome inhibitor) as a tool for the treatment of skeletal metastasis from breast cancer. The curcumin and bortezomib are loaded in the PLGA nanoparticle decorated with alendronate, which facilitated the delivery of drugs to the site of tumor-induced osteolysis. The antitumor and anti-resorptive effects were evaluated in human cell lines mimicking the bone metastatic tumors *in vitro* and in mouse models of breast cancer metastasis. The alendronate-coated nanoparticles reach the bone much faster than the non-coated nanoparticles and remained there for longer periods of time, which indicated its high bone-targeting efficiency *in vivo*. In addition, the therapeutic results *in vivo* showed that the groups treated with co-encapsulation of curcumin and bortezomib in the alendronate-coated nanoparticles significantly reduce tumor induced bone resorption and decreased the rate of tumor growth with remaining intact growth plate and greater bone structure compared to free drugs, vehicle, and untreated control groups. Therefore, these bisphosphonate-modified nanocarriers have synergistic effects in inhibition of cancer progression and osteoclastogenic activity *in vitro* and *in vivo*, which has preferential targets to bone microenvironment and prevent and treat bone metastasis [6].

In addition, alendronate (ALN) and the chemotherapeutic drug, paclitaxel (PTX), were attached to *N*-(2-hydroxypropyl)methacrylamide (HPMA) copolymer through enzyme-labile linkers. The linkers could be cleaved by cathepsin B, an overexpressed and secreted enzyme in tumor endothelial and epithelial cells [27]. The conjugate, HPMA copolymer-PTX-ALN, has significantly reduced the growth of tumors and increased the apoptotic rate of cancer cells in mice bearing mammary adenocarcinoma inoculated into the tibia. Further, the conjugate exerted an antiangiogoenic effect by decreasing microvessel density (MVD), and inducing apoptotic circulating endothelial cells (CEC) *in vivo*. The treatment of conjugate has a similar safety profile *in vivo* compared to those mice treated with saline, whereas the mice treated with free alendronate plus paclitaxel caused significant loss of body weight. The improved antitumor efficacy and decreased toxicity achieved by the conjugate resulted in specific delivery and selective release at the tumor sites. The treatment with this conjugate demonstrated improved efficacy, better tolerated safety and much simpler clinical utility than clinically used paclitaxel formulation. This conjugate would be an potential replacement of clinically used PTX formulated in Cremophor/ethanol, a formulation which is associated with a number of severe side effects, including hypersensitivity, neurotoxicity and dramatic allergic reactions [7].

However, recent research and clinical toxicity studies have reported that bisphosphonates have an inhibitory effect on osteoblastic function [28]. The excessive and prolonged suppression of bone turnover results in poor bone quality in the treatment of osteoporosis [29]. Moreover, bisphosphonates are associated with a variety of adverse events from the acute phase response, hypocalcaemia and secondary hyperparathyroidism. Furthermore, it is reported that the bisphosphonates would lead to the development of jaw osteonecrosis. Ninety-five percent of cancer patients after receiving high-dose intravenous bisphosphonates developed jaw osteonecrosis [30]. Although the causal relationship between bisphosphonates and jaw osteonecrosisis are still under investigation [31], more and more clinical investigations have shown the strong association between bisphosphonate therapy and jaw osteonecrosis [32,33]. Therefore, it is important to emphasize the prevention of jaw osteonecrosis during bisphosponate therapy.

Compared to systemic administration of targeted delivery systems, calcium phosphate biomaterial used in bone drug delivery by local implants is an alternative approach for bone regeneration and bone repair in bisphosphonate therapy. The calcium phosphate biomaterials could precipitate at low temperature *in vivo*. It is injected during surgery to minimize invasive procedures for delivering drugs in the treatment of critical size bone defects [34]. The calcium phosphate and bisphosphonate combined as the bone graft and this bone graft is injected to fill in the defects at the proximal medial tibia in rabbits. This calcium phosphate alendronate composite bone graft could be a local implant drug for increasing bone formation in the osteoporotic model [35]. Interestingly, the calcium phosphate biomaterials could effectively load the zoledronate and controllably release zoledronate to inhibit osteoclastic resorption without affecting osteoblasts *in vitro* [36]. Thus, the calcium phosphate biomaterial is a suitable carrier for delivering bisphosphonates, which is an optional choice for bone drug delivery.

##### Tetracycline

Tetracycline is another small molecular compound that could avidly chelate to calcium and has previously been evaluated as a bone tissue-targeting moiety following conjugation with therapeutic agents [37]. The tetracycline possessed numerous characteristics, such as oral bioavailability and relatively low toxicity. A novel bone tissue-targeting delivery system, the conjugation of the ring A of tetracycline with estradiol, was shown to possess significant binding affinity to the hydroxyapatite. In addition, the tetracycline-derived bone tissue-targeting agent conjugated with estradiol increased the accumulation of estradiol in the skeletal tissues of the ovariectomized rats. As a result, the model rats treated with the conjugation significantly increased femoral mass but not uterine mass, which indicated that this targeted delivery system has the potential to improve safety in the treatment of osteoporosis [8]. Tetracycline not only preferentially binds to bone, but also has high safety in bone without induction of jaw osteonecrosis. Its derivatives would be an alternative for the bisphosphonates in taking functions as a bone tissue-targeting moiety.

#### 2.1.2. Biological Molecules

Biological molecules are now attracting attention of researchers as promising targeting agents. They possess many advantages over the previously mentioned synthetic compounds, including the ease of synthesis and modification, as well as good biocompatibility. Although many biological molecules have potential to target bone at tissue level, a few were reported as targeting ligands in drug delivery systems for delivering drugs to pathological locations during treating skeletal disorders. Here, we introduce nanoparticles reported as conjugating with peptides showing bone tissue-targeting potential as nanomedicines for the treatment of skeletal disorders [38] that could provide an alternative bone-seeking moiety of the synthetic compounds.

##### Cyclic Arginine-Glycine-Aspartic Acid-Tyrosine-Lysine Peptide (cRGDyk)

Bone is the ideal site for orthotopic and metastatic tumor because its physiological environment supports tumor growth, inoculation and progress. However, the presence of a blood-bone barrier that negatively affects the penetration efficiency for anti-tumor drugs, the application of some peptides with cell penetration may overcome this problem. The cyclic arginine-glycine-aspartic acid-tyrosine-lysine peptide (cRGDyk) facilitates its cargos into targeted cells through cell membrane integrin recepetors [39]. In addition, this peptide could selectively target αvβ3 intergrin by inhibiting integrin-rich tumor cells in bone [39]. Thus, the cRGDyk conjugated with nanoparticles encapsulating cisplatin was developed to improve cellular uptake and antitumor efficiency in a murine model of bone metastasis from prostate cancer. This cRGDyk peptide conjugated liposomal drug delivery system has three fold higher cellular uptake and higher cytotoxicity to murine prostate cancer cells *in vitro* compared to mice treated with liposome encapsulating drugs. In addition, the cRGDyk peptide conjugated liposomal drug delivery system not only improved pharmacokinetic profiles of antitumor drugs, but also increased accumulation of drugs in bone through via passive targeting (EPR effects) and active targeting. This could decrease non-targeted organ toxicity and increase therapeutic efficacy *in vivo*. Thus, cRGDyk conjugated with nanoparticles could serve as an effective drug system for the synergistic targeting therapy of bone cancer and metastases [9]. Interestingly, the cRGDyk peptide could cause interference with linking the adhesion of osteoclasts to the bone matrix by blocking integrin on osteoclasts, which could be a potential therapeutic strategy inhibiting osteoclastic lesions in bone [40].

### 2.2. Cell-Specific Targeting Moiety

The skeletal systems are made up of different functional cells and ingredients. The bone tissue-targeting moieties could target the entire skeletal systems, rather than only the functional cells in bone. Non-specific drug delivery will have a high risk of adverse effects in non-targeted cells; therefore, cell-specific targeting moieties were selected. According to different targeted cells, the cell-specific targeting moieties in a targeted delivery system could be divided into three types: osteoblast-targeting, osteoclast-targeting, and bone marrow mesenchymal stem cell-specific moiety.

#### 2.2.1. Osteoblast-Targeting Moiety

Recently, there were two main methods to target osteoblasts. One method is to approach bone formation surfaces to target osteoblasts. The physical chemistry of bone-formation surfaces covered with osteoblasts is characterized by low crystallized hydroxyapatite, as well as amorphous calcium phosphate, compared to highly crystallized hydroxyapatite on bone resorption surfaces [41]. The physical and chemical difference between bone formation and resorption surfaces facilitates the design of targeting moieties. The other method is to target osteoblasts directly. The new technique of exponential enrichment (cell-SELEX) gives the hope of screening the specific cell-targeting moiety. The targeting moiety will be selected from a random pool of 10^13^ to 10^16^ ssDNA or ssRNA molecules. The potential targeting sequences, having high binding affinity to the target molecules and low binding affinity to the non-targeted molecules, will be enriched during the SELEX process. The targeted molecules are from small molecules (metal ions, organic dyes, amino acids, or short peptides) to large molecules (proteins, whole cells, viruses, virus-infected cells or bacteria). The selected moiety could bind to different targets based on their distinct three-dimensional structure without recognizing natural structures of targets [42,43]. One osteoblast-targeting aptamer has been selected to use as targeting moiety in the delivery system. Therefore, in this section, we review recent research advances in peptide and aptamer targeting moieties used in osteoblast-targeting delivery systems.

##### Tripeptide Aspartate-Serine-Serine (DSS)

The tripeptide aspartate-serine-serine (DSS) was the first designed peptide to target dentin phosphoprotein, one of the major non-collagenous proteins thought to be involved in the mineralization of the dentin extracellular matrix during tooth development [44]. This sequence of peptide has high affinity to low crystallized hydroxyapatite, the physical trait of bone formation surfaces [10]. It has verified that the DSS peptide preferred to bind bone formation surfaces rather than bone resorption surfaces *in vivo* [10]. A delivery system involving dioleoyltrimethylammonium propane (DOTAP)-based cationic liposomes conjugated with six repetitive sequences of DSS ((DSS)_6_) for delivering osteogenic siRNAs was designed to approach bone formation surfaces for targeting osteoblasts. *In vivo* systemic administration showed that this targeted delivery system would facilitate delivery of the osteogenic siRNA targeting casein kinase-2 interacting protein-1 (encoded by *Plekho1*), CKIP-1 siRNA, in osteoblasts and the subsequent depletion of specific CKIP-1 mRNA *in vivo*. In addition, the micro computed tomography (micro-CT) data indicated that the delivery system encapsulating CKIP-1 siRNA could markedly promote bone formation, enhance the bone micro-architecture and increase the bone mass in both healthy and ovariectomized rats [10]. The designed delivery system could facilitate CKIP-1 siRNA to approach bone formation for targeting osteoblasts in treating diseases marked by impaired bone formation, which is a potential strategy in clinical translation of RNAi-based bone anabolic therapies.

##### Osteoblast-Targeting Aptamer

Aptamers, the short, single-stranded DNA- or RNA-based oligonucleotides, can selectively bind to small molecular moieties or protein targets with high affinity and specificity, when folded into their unique three-dimensional structures [45]. Because of their specificity, low immunogenicity and toxicity, it has also encouraged the selection of specific cell-type aptamers and the development of aptamer-functionalized targeted drug delivery systems. Although (DSS)_6_-liposomes could specifically approach bone formation surfaces, there is still a lack of targeted delivery systems for specifically targeting osteoblasts at the cellular level. Thus, the osteoblast-specific aptamer, CH6, was screened by cell-based systematic evolution of moieties by cell-SELEX, which could specifically target both rat and human osteoblasts. The CH6 aptamer-functionalized lipid nanoparticles (LNPs) encapsulating osteogenic pleckstrin homology domain-containing family O member 1 (*Plekho 1*) siRNA promoted bone formation and microarchitecture, increased bone mass and enhanced mechanical properties in both osteopenic and healthy rodents. This osteoblast-specific aptamer-functionalized LNPs could not only act as a new RNAi-based bone anabolic strategy, but also fill the need for osteoblast-specific delivery system *in vivo* at the cellular level [11].

#### 2.2.2. Osteoclast-Targeting Moiety

Osteoclasts cooperate with osteoblasts to complete bone remodeling during a lifelong process. Abnormal increases in osteoclastic differentiation and activity results in exceeded bone resorption over bone formation, which leads to decreased bone density and increased bone fragility [46]. Skeletal disorders with dominant bone resorption, such as bone metastases and inflammatory arthritis, lead to periarticular erosions and painful osteolytic lesions. On the other hand, osteopetrosis is a rare bone disease caused by genetic mutations with decreased bone resorption, leading to accumulation of bone mass [47]. Thus, the development of osteoclast-targeting delivery systems for osteoclast-targeting therapeutics is of great significance.

##### Acid Octapeptide with Aspartic Acid

Small acidic peptides consisted of aspartic acid (Asp) were preferentially attached on the face of hydroxyapatite *in vitro*. The d-Asp_8_ has proved to preferential binding to hydroxyapatite with higher crystallinity, which is characterized by bone resorption surfaces. HPMA copolymer-d-aspartic acid octapeptide (d-Asp_8_) conjugates have been demonstrated to favorably recognize bone resorption sites in skeletal tissues [41]. Because osteoclasts and pre-osteoclasts were occupied on the bone resorption surfaces, a targeting system conjugating d-Asp_8_ peptide with liposomes for delivering microRNA modulators by specifically approaching bone resorption surfaces to target osteoclasts was developed. The delivery system could facilitate the accumulation of microRNA modulators *in vivo* and resulted in reduced bone resorption and attenuated deterioration of trabecular architecture in osteoporotic mice with no significant liver and kidney toxicity [12].

The *N*-(2-hydroxypropyl)methacrylamide (HPMA) copolymers using d-Asp_8_ as targeting moiety was linked with prostaglandin E_1_ (PGE1), a potent and well-established anabolic drug for skeletal diseases, via a cathepsin K-sensitive linkage. Because osteoclasts highly express cathepsin K, a cathepsin K-sensitive peptide linkage to attach drugs, will selectively release free drug at resorption sites during osteoclast-derived cathepsin K approaching these sites. These studies confirm the skeletal uptake of the HPMA copolymer conjugates and demonstrate that a single injection of the d-Asp_8_-FITC-PGE1 conjugate promoted bone formation in the aged ovariectomized rat model [13].

The d-Asp_8_ attached to polymeric nanoparticles with the incorporation of small interference RNA (siRNA) for semaphorin4D (sema4D), a key modulator of osteoclastic bone resorption. This system would improve the osteoclastic uptake of sema4D siRNA and intracellular trafficking within osteoclasts, which prevent the suppression of osteoblastic activity. Further, these polymeric nanoparticles specifically increased bone targeting more than three-fold when compared with controls. And the syndromes of osteoporosis in animal models induced by ovariectomy have been improved after weekly intravenous injections. The use of the hydrophilic d-Asp_8_ as both the effective targeting agent as well as the hydrophilic micelle corona was accomplished by binding of doxorubicin via an acid-sensitive hydrazine bond to achieve osteosarcoma treatment [14].

Although estrogen is widely used for estrogen substitute therapy in the treatment of osteoporosis, the large therapeutic administration would cause adverse effects, such as intrauterine hemorrhage, endometrial and breast cancers. A delivery system carrying estradiol-17β conjugated with l-Asp_6_, a targeting moiety, was developed to deliver drugs to bone. After *in vivo* injection of this delivery system, the drugs selectively distributed in bone and effectively prevented bone loss without altering the uterine weight in ovariectomized-induced osteoporotic murine model [15].

#### 2.2.3. Bone Marrow Mesenchymal Stem Cell-Specific Moieties

Bone marrow mesenchymal stem cells (BMSCs) have the potential to differentiate into various cell types, including adipocytes, chondrocytes, and osteoblasts [48,49]. Thus, there is great potential for the clinical therapeutic value of BMSCs in skeletal tissue repair and regeneration. The targeted drug delivery to BMSCs has influence on their differentiation and proliferation.

##### Bone Marrow Mesenchymal Stem Cell-Specific Aptamer

To achieve the BMSC-specific targeting, an apamter targeting BMSCs was screened by cell-SELEX. The BMSC-specific aptamer was linked to antagomir-188, which could specifically inhibit high levels of miR-188 in BMSCs from the aged mice. The age-related increase in miR-188 functions as a switch to regulate BMSC differentiation between osteogenesis and adipogenesis, which indicates the miR-188 will be a potential therapeutic target for the age-related bone loss. The therapeutic efficiency of conjugation in aged mice was stimulated, with increased bone formation and decreased bone marrow fat accumulation after intra-bone marrow administration. This indicated a potential therapeutic to target BMSCs with regulating miR-188 for treatment of age-related bone loss [50].

### 2.3. Potential Targeting Moieties

Some targeting moieties have been discovered recently. However, they have not been applied in the development of targeted delivery systems. In this section, we summarize some newly founded targeting moieties with potential use for targeted delivery systems in the therapeutics of skeletal disorders.

#### Pseudopurpurin

The red-colored bone has found in some Guishan goats. Only one compound, pseudopurpurin, was extracted and identified from those red-colored bones by liquid chromatography-mass spectrometry (LC-MS). This result indicated that pseudopurpurin is a potential targeting moiety to bone. Interestingly, the results from micro-CT showed that the red-boned goats displayed an increase in the trabecular volume fraction, trabecular thickness, and the number of trabeculae in the distal femur compared to those of common goats. To further confirm the effect of pseudopurpurin on bone geometry, architecture, and metabolism, the rats were fed diets with added pseudopurpurin. Similar changes were observed in the femurs of the rats after one, three and five months of pseudopurpurin feeding compared to the non-treated rats. In addition, the mRNA expression of alkaline phosphatase (ALP), bone GLA-protein (BGP) in the rats was significantly higher in the rat treated with pseudopurpurin than the same-age controls. The receptor activator of nuclear factor kappa-B ligand (RANKL) mRNA in the treated rats decreased significantly after five months of feeding compared with the same-age controls. The above results demonstrate that pseudopurpurin has not only a close affinity to bone, but also a high level of mineral salts in the bone leading to improvement in bone strength and enhancement in the structure and metabolic functions of the bone [51]. The chemical structure of pseudopurpurin is similar to alizarin, a high-affinity chemical to bone and would have a selective affinity for the principal salts of bone, the pseudopurpurin-calcium salt. The salts will come into contact with osteoid-tissue and the organic component of developing bone. Therefore, the pseudopurpurin-calcium salt has an adhesiveness to calcium ions preventing their loss in bone metabolism [16].

## 3. Nanoparticles

In recent years there has been an unprecedented growth in studies and applications in the area of nanoscience and nanotechnology in targeted delivery of medicines. Nanoparticles would improve water-solubility, control the release rate, and reduce toxicity of drugs. Because of their unique features in drug delivery, nanoparticles, such as liposomes, lipid nanoparticles (LNPs), and poly(lactic-*co*-glycolic acid) nanoparticles, are widely used in the fields of molecular therapies in bone regeneration, fracture repair and bone cancer (Table 2). In addition, flexible nanoparticles also are able to carry medicines through blood vessels and bone sinusoids, and the stealth nanoparticles extend their circulation in the blood stream to reach pathological location, evading detection in immune systems [52,53]. Consequently, they facilitate the targeting of drugs into different types of cells in bone and various cellular compartments, including the nucleus.

Moreover, nanoparticles have been approved by the FDA for use in treating cancers. Similarly, the clinical application of nanoparticles in skeletal disorders is also feasible. To achieve the desired clinical application, nanoparticles should be nontoxic, with low immune reactivity, biodegradable and effective. In this section, several representative nanoparticles for molecular delivery applied in skeletal disorders are highlighted.

### 3.1. Liposomes

The concept of liposomes, the closed bilayer phospholipid systems, was first described in 1965. In its 50 years of development, a number of products have been sold on the market, such as AmBisome^®^, Doxil^®^ and Marqibo^®^, with many more in clinical development. The “first-generation liposomes” overcame problems such as low drug load and uncontrollable rate of the drug release. However, the rapid clearance of the first-generation liposomes by the mononuclear phagocyte system (MPS) not only influenced drug delivery and therapeutic efficiency, but also increased toxicity to the MPS organs [55,56]. To solve this problem, the “second-generation liposomes”, long-circulation liposomes, were developed by modulating the lipid compositions, particle sizes and charges of liposomes. A significant improvement of long-circulating liposomes was inclusion of the artificial polymer poly-(ethylene glycol) (PEG) in liposome. The shield of PEG would avoid the uptake of liposomes by the MPS [57]. Particularly, the long-circulating liposomes modified with alendronate were developed to carry ^99m^technetium-ceftizonxime. It exhibited higher uptake in regions of septic inflammation, which would be used in identification of osteomyelitis [58]. In addition, liposomes containing polyethylene glycol encapsulated cisplatin (CDDP-L) were prepared to encapsulate caffeine, which remain in the systemic circulation for a long time and accumulated in a rat osteosarcoma model [59].

Because the MPS will take up the delivery system, researchers took advantage of this mechanism to develop a bone marrow-targeted delivery system for diagnostic and therapeutic reasons. Mononuclear phagocyte system (MPS) organs have sinusoids with a fenestrated endothelium in association with macrophages, which express several types of receptors on their surface for the uptake of specific nanoparticles. The bone marrow is a part of the MPS. Thus, the nanoparticles in the blood circulation would be taken up by the MPS and gradually go to bone marrow [60]. The liposomes to target bone marrow are made up of four kinds of lipids, 1,2-Dipalmitoyl-*sn*-glycero-3-phosphocholine (DPPC), cholesterol (CH), 1,2-distearoyl-*sn*-glycero-3-phosphoethanolamine-*N*-[monomethoxy poly(ethylene glycol) (5000)] (PEG-DSPE) and l-glutamic acid, *N*-(3-carboxy-1-oxopropyl)-1,5-dihexadecyl ester (SA). Although the utility of PEG would reduce the uptake of MPS, the liposome size could modulate the stealth property of PEG [61]. In addition, the nanoparticle would trigger a clearance phenomenon on particle size-dependence [62]. Due to the particle size in the range of 200–270 nm, the liposomes to target bone marrow would escape from liver and spleen uptake but specifically deliver technetium-99m (^99m^TC), a metastable nuclear isomer as medical radioisotope, to the bone marrow after intravenous administration in rabbits. This design would open up a wide variety of new therapeutic applications [63].

### 3.2. Lipid Nanoparticles

Lipid nanoparticles (LNPs) have been widely used for medicines, due to their lipid biocompatibility and versatility. Unlike liposomes in the same category, LNPs show considerable kinetic stability and rigid morphology. Their low cytotoxicity, production scalability, the modulation of drug release, the avoidance of organic solvents, and wide potential application spectrum is greater than liposomes [64]. In addition, LNPs overcome some current challenges in drug delivery and therapy. The LNPs have a smaller nanoparticle size, from 1 to 100 nm, compared to the average diameter of liposomes in the range from 400 nm to 2.5 µm. The small size of LNPs facilitate the drugs to go through bone sinusoids. Moreover, the size of LNPs could prevent the detection by MPS in blood circulation and reduce the accumulation in spleen and liver.

In addition, the emergence of therapeutic siRNA and microRNA provides a novel avenue in molecular therapy of skeletal disorders. However, the safety, efficiency and targeted delivery of therapeutic siRNA and microRNA *in vivo* are still obstacles in their clinical translation. siRNA and microRNA are hydrophilic negatively charged macromolecules, very labile in biological fluids: the systemic administration of naked siRNAs or microRNA does not result in effective therapeutic responses. Then, the interaction between active molecules and biological membranes is necessary to initiate the entrance into cells, but this process is not spontaneous because the negatively charged surface of siRNA and microRNA and cell membranes hampers the interaction, and their hydrophilic character prevents the passing through lipophilic cell membranes. But the LNPs can carry large amount of siRNAs and microRNA to enter into cells by endocytosis [65]. The LNPs with the ideal size (1–100 nm) to escape from the MPS uptake and easily cross the bone sinusoids are a perfect partner in gene delivery for skeletal therapies [66]. The stable nucleic acid lipid particles (SNALP) is a family of the LNPs. The ionizable cationic lipid, 2,2-Dilinoleyl-4-(2-dimethylaminoethyl)-[1,3]-dioxolane (DLin-KC2-DMA), is a key lipid component of SNALP. The lipid was applied into osteoblast-targeting aptamer-functionalized nanoparticles to specifically deliver osteogenic CKIP-1 siRNA into osteoblasts [11]. This application of the LNPs in targeted delivery of osteogenic CKIP-1 siRNA provides a promising RNA interference-based bone anabolic strategy.

### 3.3. Poly(Lactic-co-Glycolic Acid) Nanoparticles

Poly(lactic-*co*-glycolic acid) (PLGA) is one of the most successfully developed biodegradable polymers. The PLGA has attracted considerable attention due to its attractive properties. It has been approved by the FDA and the European Medicine Agency as a drug delivery system with characters of biodegradability, biocompatibility, well-described formulations, various types of drug encapsulation and sustained release. PLGA is made up of lactic acid and glycolic acid, which would be easily metabolized by the body via the Krebs cycle. Thus, there is a minimal systemic toxicity associated with the use of PLGA for drug delivery or biomaterial applications [67].

PLGA could be used in treatment of inflammatory diseases because the PLGA-based drug delivery systems are preferentially taken up by the MPS and lead to high and selective accumulation in inflamed areas. This phenomenon might be explained by increased presence of immune-related cells like macrophages, lymphocytes or dendritic cells (DCs) and by disruption of epithelium in these inflamed sites, resulting in a preferential accumulation of these nanoparticles. In addition, the use of PLGA could not induce “crystal-induced pain” because of its biodegradability, biocompatibility and small size. The PLGA nanoparticle encapsulated glucocorticoids were directly injected inside the rat joint cavity. The results showed that the PLGA nanoparticles were preferentially phagocytozed by macrophages, which would specifically deliver glucocorticoids to the inflamed cells. It could also facilitate the medicines remaining in synovium, which is the pathological site of rheumatoid arthritis [68].

The PLGA nanoparticles modified with poly aspartic acid peptide (poly-Asp) were designed as a bone-tissue targeting delivery system. This bone-targeting delivery system has strong affinity to high hydroxyapatite (HA), which is characterized by the bone resorption surfaces [54]. It indicated that poly-Asp could facilitate the PLGA nanoparticles specifically delivering drugs through approaching bone resorption surfaces to target osteoclasts.

## 4. Challenges and Perspectives

Since the concept of targeted drug delivery systems appeared in 1906, this concept has been gaining much attention and introduced as therapeutic approaches in skeletal disorders gradually. In recent years, the bone-seeking moieties were discovered and applied in targeted delivery system of molecular therapy to enhance and prolong pharmacological effects in bone, reduce side effects in non-targeted issues, and improve compliance because of less frequent need for medication. Previously, we gave more attention on the targeting moieties for bone tissues, which is an important part in various bone-targeting delivery systems. However, bone tissues are made up of different type of cells, such as osteoclasts, osteoblasts, bone marrow mesenchymal stem cells, and osteocytes*.* The bone tissue-targeting delivery systems deliver drugs to the whole skeletal tissues, not the specific functional cells in bone, which reduce the efficiency and increase toxicity on neighboring non-targeted cells during treatment. In addition, some targeting moieties, such as pamidronate, alendronate and tiludronate, have dual effects on osteoblasts and osteoclasts and potential induction of jaw osteonecrosis. Moreover, one of alternative approaches in the clinic for bone regeneration and bone repair is calcium phosphate biomaterials [69]. This method is widely used as osteoinductive biomaterials by local implants. This is an alternative choice in administration of active agents compared with systemic treatments.

The new generation of targeting moieties, such as peptides and aptamers, appeared to improve the accuracy of targeting in order to increase the therapeutic efficiency and reduce adverse effects to the untargeted cells. The most important one is the aptamer, which could exhibit cell-type specific seeking and exert the modulation of specific functional cells in bone. The emergence of cell-SELEX technique will allow a big stride forward to screen specific cell-type targeting aptamers to bone. The osteoblast-targeting aptamer has been screened as targeting moieties in the osteoblast-targeting delivery systems. Although BMSCs-specific aptamers have been used as targeting ligands in the conjugate, the administration of conjugate by intra-bone marrow will limit its utility. In addition, it is still lacks targeting moieties for cartilage cells and osteoclasts. With the development of this technique, these drawbacks of targeted delivery systems in of skeletal therapy could fill up in the near future.

The conventional nanoparticles have their own limitation, which drove the nanoparticles for molecular therapy in skeletal diseases to upgrade, to become biostable, biocompatible and biodegradable. The nanoparticles become an important and significant partner to bone-seeking agents during the development of targeted delivery system in skeletal diseases. The components and the size of nanoparticles are two important factors during the development of targeted delivery systems in skeletal therapy. The use of polyethylene glycol (PEG) in nanoparticles could avoid the clearance of the MPS to some extent, which protects drugs from degradation before arrival to the specific pathological sites. However, limited therapeutic efficacy and loss of their long circulating property of PEG-conjugated nanoparticles have been recently reported in animals and humans following repeated injection due to the induction of anti-PEG antibody [70]. Thus, the novel type of ingredients used in targeted delivery systems should be under investigation. Moreover, because the size of bone sinusoids is less than 80 nm, the size of nanoparticles should be smaller than bone sinusoid to guarantee the efficiency of drug delivery. In addition, the nanoparticles themselves would not have a harmful effect to diseases, but the derivatives or metabolites might influence therapeutic efficiency and aggravate damage during the treatment. For example, the PLGA nanoparticle is the most of successful delivery systems, which has been approved by the FDA and European Medicine Agency. Some results showed that the metabolites of the PLGA nanoparticle are acidic chemicals with potential harmful impacts on the inflammatory bone tissues. These results indicate that the ingredients of nanoparticles influence the efficiency of targeted delivery during treatment. When we establish a nanoparticle, we should consider more than targeted delivery but also the characters of lipid ingredients and their metabolites. Although the metabolites of the PLGA nanoparticle would influence the recovery of inflammatory bone tissues, it has been used in the treatment of rheumatoid arthritis. This implies that we should make thorough studies of nanoparticles before making decisions. More and more research focuses on the new generation of targeted delivery systems, which contain conditional fracture linkers between nanoparticles and targeting moieties. Some of them are pH-responsive or enzyme-responsive [71,72]. The emergence of new nanoparticles would accompany the development and improvement of materials.

## 5. Conclusions

Although many challenges for targeted delivery systems of molecular therapy in skeletal disorders have come to the forefront, such as manufacture cost, biostability, pharmacokinetic and therapeutic efficiency, the therapeutic strategy of targeted delivery systems used in skeletal diseases still have attractive advantages. The evaluation of targeted delivery systems in skeletal diseases requires complicated and costly safety and efficacy experiments, but the engineering of this targeted delivery system is fairly simple owing to cost-effective, and easy manufacture. Thus, the targeted delivery systems for molecular therapy in skeletal disorders have an immense clinical potential leading to a new generation of disease treatment. The recent exploration of various targeted delivery systems for molecular therapy in skeletal disorders is worthy of note.

## Figures and Tables

**Table 1:** Examples of targeting moieties used in targeted delivery systems for molecular therapy in skeletal disorders.

Targeting Moieties	Targeted Tissues or Cells	Drugs Delivered	References
Bisphosphonate	Skeletal tissues	Curcumin, bortezomib and paclitaxel	[6,7]
Tetracycline derivate	Skeletal tissues	Estradiol	[8]
cRDGyk	Integrin-rich tumor cells	Cisplatin	[9]
(DSS)_6_	Bone formation surfaces	CKIP-1siRNA	[10]
CH6	Osteoblasts	CKIP-1siRNA	[11]
d-Asp_8_	Bone resorption surfaces	microRNA modulator, PGE1 and sema4D siRNA	[12,13,14]
l-Asp_6_	Bone resorption surfaces	Estradiol-17β	[15]
BMSCs-specific aptamer	BMSCs	Antagomir-188	[16]

BMSCs: Bone marrow mesenchymal stem cells.

**Table 2:** Examples of nanoparticles used in targeted delivery systems for molecular therapy in skeletal disorders.

Nanoparticles	Targeting Moieties	References
Liposome	cRGDyk, (DSS)_6_ and d-Asp_8_	[9,10,12]
LNPs	CH6	[11]
PLGA nanoparticle	Bisphosphonate, poly-Asp	[6,54]

LNPs: lipid nanoparticles; PLGA: poly(lactic-*co*-glycolic acid).
